# Constraint Programming Based Biomarker Optimization

**DOI:** 10.1155/2015/910515

**Published:** 2015-05-05

**Authors:** Manli Zhou, Youxi Luo, Guoquan Sun, Guoqin Mai, Fengfeng Zhou

**Affiliations:** ^1^Shenzhen Institutes of Advanced Technology, Chinese Academy of Sciences, Shenzhen, Guangdong 518055, China; ^2^Shenzhen College of Advanced Technology, University of Chinese Academy of Sciences, Beijing 100049, China; ^3^School of Science, Hubei University of Technology, Wuhan, Hubei 430068, China

## Abstract

Efficient and intuitive characterization of biological big data is becoming a major challenge for modern bio-OMIC based scientists. Interactive visualization and exploration of big data is proven to be one of the successful solutions. Most of the existing feature selection algorithms do not allow the interactive inputs from users in the optimizing process of feature selection. This study investigates this question as fixing a few user-input features in the finally selected feature subset and formulates these user-input features as constraints for a programming model. The proposed algorithm, fsCoP (feature selection based on constrained programming), performs well similar to or much better than the existing feature selection algorithms, even with the constraints from both literature and the existing algorithms. An fsCoP biomarker may be intriguing for further wet lab validation, since it satisfies both the classification optimization function and the biomedical knowledge. fsCoP may also be used for the interactive exploration of bio-OMIC big data by interactively adding user-defined constraints for modeling.

## 1. Introduction

Biological big data is being accumulated at an accelerated speed, facilitated by the rapid invention and development of bio-OMIC data production technologies [1, 2]. Interactive exploration technology is widely used to mine knowledge from various big data areas [3] and may be useful to rapidly and accurately detect phenotype-associated biomarkers from the huge amount of bio-OMIC data [4]. This is usually formulated as the feature selection problem [5, 6].

Various algorithms have been proposed to choose a few from a large number of features, by optimizing a phenotypic measurement. The principle of parsimony prefers a minimum number of features for an accurate representation of the data [7]. Detailed introduction may be found for both general feature selection algorithms [8] and phenotype-associated biomarker detection algorithms [9] from the literature. Considering millions or more of bio-OMIC features for each sample, although the exhaustive search guarantees the detection of optimal feature subset, its computational requirement exceeds the capacity of any high-performance computing systems under the current parallel computing architecture. So all the existing feature selection algorithms screen for the suboptimal solutions based on some heuristic rules.

Heuristic feature selection algorithms may be grouped as two classes based on how they generate the finally chosen features. The class I wrapper or group optimization algorithms evaluate a feature subset by testing its classification performance with a learning algorithm. The features are selected by heuristic rules or randomly, and only the feature subset with the best classification performance will be kept for further investigation, for example, forward stepwise selection [10] and ant colony optimization [11]. The class II filtering or individual ranking algorithms measure each feature's correlation with the class labels and rank the features by their measurement. A heuristic assumption is that the combination of top-ranked *K* features should produce a good classification performance, where *K* is an arbitrarily chosen integer. They are usually much faster than the class I algorithms but lack model robustness due to the ignorance of feature interdependence [12]. It is also difficult to determine how many features should be chosen from the ordered feature list.

This work proposes a constraint programming based interactive feature selection algorithm, fsCoP, for efficient exploration of the bio-OMIC big data. An interactive feature selection problem requires a fast and accurate detection of features and the integration of user-input features in the final result. The majority of existing feature selection algorithms do not consider how to make sure a given feature subset appears among the finally selected features. fsCoP fixes the user-input features in the result by formulating them as constraints of the programming model. Our data show that features chosen by fsCoP perform well similar to or much better than the existing feature selection algorithms in classification, even with the constraints of fixed features from both literature and other algorithms.

## 2. Materials and Methods

### 2.1. Dataset Downloading and Preprocessing

Two microarray-based gene expression profiling datasets are downloaded from the NCBI GEO database [13]. Both datasets GSE5406 [14] and GSE1869 [15] profiled ischemic cardiomyopathy samples and their controls on the Affymetrix Human Genome U133A Array (HG-U133A) platform. The transcriptomes are normalized using the RMA algorithm [16]. The gene expression profiles of ischemic cardiomyopathy samples and the nonfailing controls are kept for binary classification study in this work.

### 2.2. Feature Selection Based on Constraint Programming (fsCoP)

This work proposes a constraint programming based feature selection algorithm, allowing the user to determine a few features in the finally chosen feature subset. The prefixed features may be the biomarkers known to be associated with the phenotype in the literature or the features selected by other feature selection algorithms. This model is proposed to answer the biological questions like whether a few genes together with the ischemic cardiomyopathy associated ACE2 (angiotensin-converting enzyme-2) may constitute an accurate model for the disease early detection. The majority of the existing feature selection algorithms do not have the integrating component for fixing a few features in the final feature subset. Let FixedSubset be the set of features to be fixed in the final result and let *c* be the class number. Class *j* has *n*_*j*_ samples, where *j* = 1,2, …, *c*. The programming model is defined as follows:

(1)  min⁡wi,ξk  ∑i=1pwi+λ∑j=1c ∑k=1njξk,

(2)  s.t.  ∑i=1pskij−mijwi−ξk<∑i=1pskij−mi~jwi,    for  k∈1,2,…,nj, j∈1,2,…,c,

(3)     wf≥MinWeight, for  f∈FixedSubset,

(4)  wf≥MinWeight0≤wi≤1,

(5)  wf11≥MinWeightξk≥0.

The average value of the *i*th feature is denoted as *m*_*i*_^*j*^ for the samples in class *j*. Formula (2) makes that the centroid of class *j* is the closest centroid to the samples of the class *j*. Each prefixed feature has the weight no smaller than MinWeight. Each feature has a weight *w*_*i*_ ∈ [0,1], where only features with positive weights are selected by the algorithm.

### 2.3. Classification Performance Measurements

A binary classification model is trained over the datasets of positive and negative samples, whose numbers are *P* and *N*, respectively. The classification performance is usually measured by the sensitivity Sn = TP/(TP + FN) and specificity Sp = TN/(TN + FP), where TP, FN, TN, and FP are the numbers of true positives, false negatives, true negatives, and false positives. The overall classification performances may be measured by the overall accuracy Acc = (TP + TN)/(TP + FN + TN + FP) and balanced overall accuracy Avc = (TP + TN)/(TP + FN + TN + FP). Matthew's Correlation Coefficient is also calculated to measure how well a classification model is, and it is defined as MCC = (TP × TN − FP × FN)/sqrt((TP + FP)×(TP + FN)×(TN + FP)×(TN + FN)), where sqrt(*x*) is the squared root of *x*.

Fivefold cross validation (5FCV) strategy is used to train the model and calculate how well a model performs. Fluctuation may occur for different seeds of the random number generator. So 30 runs of the 5FCV experiments are carried out with different random seeds.

### 2.4. Comparison with Four Feature Selection Algorithms

The proposed feature selection algorithm fsCoP is compared with two ranking algorithms, that is, *t*-test (TRank) [17] and Wilcoxon test (WRank) [18], and two other widely used algorithms, that is, prediction analysis of microarrays (PAM) [19] and regularized random forest (RRF) [20].

The ultimate goal of the proposed model is to select a subset of features with accurate classification performance. The performance of a given feature subset is measured by five widely used classification algorithms, including support vector machine (SVM) [21], Naive Bayesian [22], decision tree (DTree) [23], Lasso [24], and *K*-nearest neighbor [25]. The classification model with the best Matthew's Correlation Coefficients is kept for the comparison study.

This work uses the default parameters of all the investigated algorithms implemented in the statistical software R/Rstudio version 3.1.1 released on July 10, 2014 [26, 27]. A classification model is usually obtained by trying multiple classification algorithms [28, 29]. So this work compares the feature selection algorithms based on the highest MCC values of the five aforementioned classification algorithms.

## 3. Results and Discussion

### 3.1. Constrains from the Literature

The angiotensin-converting enzyme-2 (ACE2) at the location Xp22.2 of the human genome HG19 is chosen to be fixed in the algorithm fsCoP, denoted as fsCoP(ACE2). ACE2 was observed to be differentially expressed between ischemic and nonischemic cardiomyopathy and may play a role in transducing the signal of heart failure pathophysiology [15]. The expression level of ACE2 is detected by two probe sets (219962_at and 222257_s_at) in the Affymetrix microarray platform U133A (GPL96). These two features will be fixed in the feature subset fsCoP(ACE2), and the performances of the five classification algorithms are compared using the selected features by fsCoP and fsCoP(ACE2).

Firstly, fsCoP and fsCoP(ACE2) achieve similarly good performance on the two investigated datasets, that is, GSE5406 and GSE1869.  Table 1 shows that, except the decision tree algorithm on the dataset GSE1869, there are no greater than 0.021 differences in MCC between the two versions of fsCoP. The greatest difference occurs for the NBayes classification algorithm on the dataset GSE1869, where fsCoP(ACE2) (1.000 in MCC) improves fsCoP (0.979).

Secondly, if only the best classification algorithm is chosen for each subset of selected features, fsCoP(ACE2) also performs well similar with fsCoP. SVM(fsCoP) only improves NBayes(fsCoP(ACE2)) by 0.001 in MCC. The other classification performance measurements also show that this is a minor improvement, with the maximal difference being 0.002 in specificity (Sp). The comparison of the best classification models between the two datasets in Table 1 also shows that NBayes(fsCoP(ACE2)) even performs 0.002 better than KNN(fsCoP) on the dataset GSE1869.

fsCoP runs fast similar with or without fixing a few features. The running time of the algorithm fsCoP with or without fixing user-selected features is compared between fsCoP and fsCoP(ACE2). Since fsCoP runs very fast, we repeat the model testing for multiple times with different random seeds, as in Table 2. The data suggests that fsCoP(ACE2) runs slightly faster than fsCoP for most of the times, except for the case of 10 repeats.

### 3.2. Comparison of fsCoP(ACE2) with the Existing Feature Selection Algorithms

**Figure 1:**
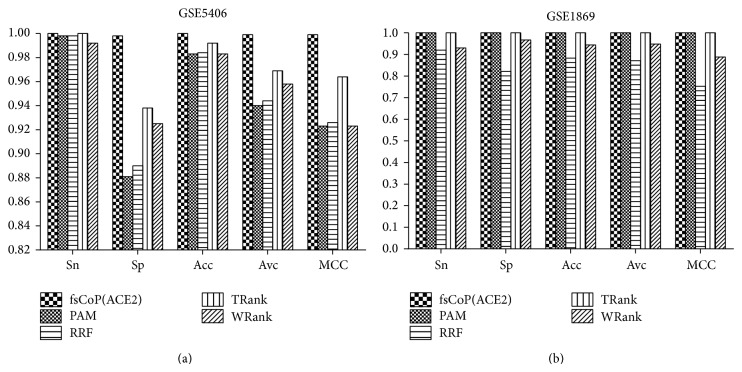
Classification performance comparison of the five feature selection algorithms on the datasets: (a) GSE5406 and (b) GSE1869. The histograms give the detailed values of the classification performance measurements, that is, Sn, Sp, Acc, Avc, and MCC.

A further comparison of fsCoP(ACE2) with the other existing feature selection algorithms is conducted for the best classification algorithms on each of the selected features, as shown in Figure 1. First of all, fsCoP(ACE2) performs the best (100%) in sensitivity (Sn) with the classification algorithm NBayes on both datasets, as in Figures 1(a) and 1(b). SVM(TRank) achieves the same sensitivities for both datasets, and KNN(PAM) also achieves 100% in Sn on the dataset GSE1869. NBayes(fsCoP(ACE2)) achieves 0.998 in specificity (Sp) on the dataset GSE5406, and no other feature selection algorithms reach the same specificity level.  Figure 1(a) suggests that the second best feature selection algorithm may be TRank, which achieves 0.964 in MCC on the dataset GSE5406.

### 3.3. Constraints from the Existing Feature Selection Algorithms

**Figure 2:**
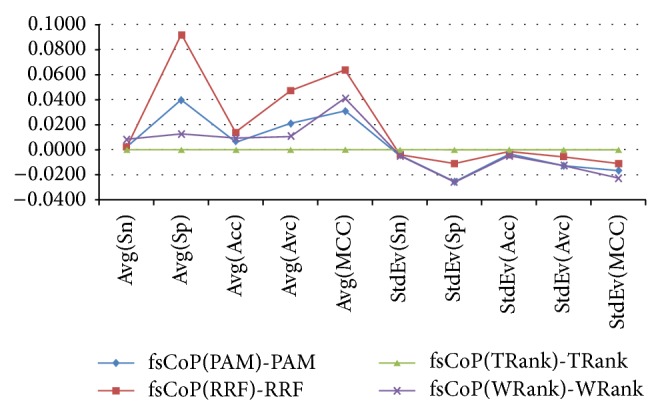
Improvements of fsCoP compared with the four investigated feature selection algorithms, by fixing the features selected by each algorithm. The average “Avg()” and standard deviation “StdEv()” of the five classification performance measurements, that is, Sn, Sp, Acc, Avc, and MCC, are calculated over the 30 runnings of 5-fold cross validations of a given feature subset.

Except for the features selected by TRank, fsCoP improves all the other three feature selection algorithms. fsCoP(A) is defined to be feature list selected by fsCoP, with the fixed features selected by Algorithm A.  Figure 2 shows that fsCoP(TRank) achieves the same classification performance as TRank, and, for the three other feature selection algorithms, fsCoP() achieves higher averaged values and smaller standard deviations for all the five classification performance measurements. The most significant improvement of fsCoP is observed for the RRF algorithm, with 0.0916 in Sp improvement. So besides the integration of known biomarkers from the literature, fsCoP may also be used to further refine the feature subset selected by the existing feature selection algorithms. Better classification performance with smaller fluctuation may be obtained stably by fsCoP, compared with the algorithms.

After the further refining by fsCoP, features selected by all the four feature selection algorithms achieve 100% in the classification sensitivity, while maintaining at least 92% in specificity. And at least 0.95 in MCC is achieved for all the four cases.

## Figures and Tables

**Table 1:** Performance comparison of the algorithm fsCoP. fsCoP has no prefixed features, and the model fsCoP(ACE2) has two predetermined features.

GSE5406					

fsCoP	Sn	Sp	Acc	Avc	MCC

SVM	**1.000 **	**1.000 **	**1.000 **	**1.000 **	**1.000 **
NBayes	1.000	0.998	1.000	0.999	0.999
DTree	0.992	0.800	0.967	0.896	0.848
Lasso	0.999	0.900	0.987	0.950	0.939
KNN	1.000	0.871	0.983	0.936	0.923

fsCoP(ACE2)	Sn	Sp	Acc	Avc	MCC

SVM	1.000	0.996	0.999	0.998	0.998
NBayes	**1.000 **	**0.998 **	**1.000 **	**0.999 **	**0.999 **
DTree	0.993	0.796	0.967	0.894	0.847
Lasso	1.000	0.907	0.988	0.953	0.944
KNN	0.999	0.860	0.982	0.930	0.916

GSE1869					

fsCoP	Sn	Sp	Acc	Avc	MCC

SVM	1.000	0.955	0.983	0.978	0.965
NBayes	1.000	0.972	0.990	0.986	0.979
DTree	0.907	0.000	0.567	0.453	NaN
Lasso	0.960	0.989	0.971	0.974	0.943
KNN	**1.000 **	**0.994 **	**0.998 **	**0.997 **	**0.996 **

fsCoP(ACE2)	Sn	Sp	Acc	Avc	MCC

SVM	1.000	0.939	0.977	0.970	0.953
NBayes	**1.000 **	**1.000 **	**1.000 **	**1.000 **	**1.000 **
DTree	0.987	0.000	0.617	0.493	NaN
Lasso	0.990	0.967	0.981	0.978	0.962
KNN	**1.000 **	**1.000 **	**1.000 **	**1.000 **	**1.000 **

**Table 2:** Running time of fsCoP and fsCoP(ACE2) on GSE5406. All the running times are calculated in seconds and column “repeat” gives the number of repeats of each model with different random seed.

Repeat	fsCoP	Avg (fsCoP)	fsCoP(ACE2)	Avg (fsCoP(ACE2))
5	11.95	2.39	11.78	2.36
10	23.83	2.38	23.96	2.40
50	120.01	2.40	117.79	2.36
100	240.23	2.40	236.75	2.37
